# Poly(I:C) and R848 adjuvants elicit sizeable humoral immunity to liver stage malaria antigens

**DOI:** 10.3389/fimmu.2026.1705839

**Published:** 2026-03-30

**Authors:** Nikunj Tandel, Mansi Thakkar, Mansi Ahuja, Jagrut Shah, Devangkumar D. Trivedi, Sylviane Pied, Agam P. Singh, Chirag Prajapati, Rajeev K. Tyagi, Sarat K. Dalai

**Affiliations:** 1 Institute of Science, Nirma University, Ahmedabad, Gujarat, India; 2 Center for Infection and Immunity of Lille-9 CIIL, CNRS UMR 9017-INSERM U1019, University de Lille, Institut Pasteur de Lille, Lille, France; 3 Infectious Disease Laboratory, National Institute of Immunology (NII), New Delhi, India; 4 Department of Biotechnology, Veer Narmad South Gujarat University (VNSGU), Surat, Gujarat, India; 5 Division of Cell Biology and Immunology, Biomedical Parasitology and Translational-immunology Lab, CSIR-Institute of Microbial Technology (IMTECH), Chandigarh, India

**Keywords:** B cell, cross-presentation, malaria, *Pb*SLTRiP, *Pf*CSP, Poly(I:C), T cell

## Abstract

**Background and objectives:**

*Plasmodium falciparum* infection remains a major global health burden due to increasing drug resistance and lack of an effective vaccine. Targeting the exoerythrocytic liver stage (LS) of malaria infection offers a promising approach to achieve sterile immunity to malaria infection. Therefore, the present work investigates the immunogenic potential of two key LS malaria antigens: circumsporozoite protein of *P. falciparum* (*Pf*CSP) and liver stage-expressed tryptophan-rich protein of *P. berghei* (*Pb*SLTRiP) formulated with a dual toll-like receptor (TLR) adjuvant combination, Poly(I:C) and R848, to evaluate the potency and durability of the elicited humoral response.

**Methods:**

BALB/c and C57BL/6 mice were immunized using a multidose regimen with *Pf*CSP and *Pb*SLTRiP, either alone or in combination (Poly(I:C) and R848). Humoral immune responses were quantified 30–80 days post-immunization through total IgG kinetics, functional IgG isotypes (IgG1, IgG2c/IgG2a), and antibody avidity (AI).

**Results:**

Coadministration of Poly(I:C) and R848 was shown to evoke greater antibody responses compared to antigen alone. *Pf*CSP-specific IgG titers remained detectable up to 80 days post immunization and declined gradually over time. *Pb*SLTRiP immunization, however, produced progressive increases in total IgG, detectable IgG2c responses, and modest changes in antibody avidity, with little non-significant difference between adjuvant regimens.

**Conclusions:**

Combination of Poly(I:C) and R848 adjuvant induced detectable antibody responses against LS malaria antigens, with magnitudes generally like those obtained with single adjuvants. Limited statistical differences highlight preliminary trends warranting future investigations into predicted multi-epitope engagement and hypothesized B cell-mediated CD8^+^ T-cell cross-presentation.

## Introduction

Malaria remains one of the most persistent infectious diseases worldwide, causing significant morbidity and mortality, particularly in tropical and subtropical regions (1). Despite efforts forwarded to control malaria infection, treatment and eradication remain challenging due to the emergence of drug and insecticide resistance (2, 3). Drug and insecticide resistance begets the need for an effective and broadly protective vaccine targeting early-stage malaria infection (2). Targeting different parasite stages could be a viable approach to develop effective vaccines (4). However, targeting the asymptomatic liver stage (LS) of infection has emerged as a promising strategy to achieve sterile immunity (5). This could be crucial, as evoked LS immune response may potentially prevent parasite progression to the symptomatic asexual blood stage of *Plasmodium* infection.

LS malaria antigens are considered critical vaccine targets since protection at this stage primarily relies on CD8^+^ T-cell responses (6–12). CD8^+^ T-cell responses must persist in the liver to eliminate infected hepatocytes. However, despite progress, current WHO-recommended CSP-based subunit vaccines, such as RTS,S and R21, achieve only short-lived efficacy and protection that wanes over time (13), whereas R21/Matrix-M has shown improved immunogenicity (14, 15). These observations are attributed to the insufficient induction of long-lasting CD8^+^ T-cell responses and reliance on relatively short-lived antibody-mediated protection. Thus, this underscores the need for next-generation subunit vaccines that elicit durable humoral and cellular immune responses (16).

Subunit vaccines, despite their excellent safety profile, often require potent adjuvants to enhance immunogenicity and achieve a sustained protective response against intracellular pathogens (17, 18). Hence, we explored a combination of two toll-like receptor (TLR) agonists-poly(I:C) (TLR3 agonist) and R848 (TLR7/8 agonist)—to boost the immunogenic potential of key LS malaria antigens. Poly(I:C) is known to favor a Th1-biased immune response, while R848 efficiently activates antigen-presenting cells (APCs) and induces cytokine profiles for T-cell priming (19–21). Therefore, simultaneous activation of these two innate immune pathways would synergistically enhance Th1 development, optimize APC maturation, and improve antigen cross-presentation on MHC class I molecules to promote effective CD8 T-cell priming is hypothesized.

Recently, the role of B cells as non-canonical APCs has been recognized as crucial in prolonging the immunity following the repeated immunization (22). Published work from our group demonstrated that repeated immunization with Poly(I:C) and R848 in an ovalbumin (OVA) model elicited balanced humoral and cellular immune responses (23).

Collectively, the present work explored the potential of a dual-adjuvant approach to induce durable and functionally active antigen-specific humoral responses against two pivotal *Plasmodium* liver-stage antigens, *Pf*CSP and *Pb*SLTRiP (24). Our data suggest that dual adjuvants are associated with a trend toward humoral breadth and persistence (transient IgG2a/c responses) and pave the way for further investigations for developing effective liver-stage malaria vaccine.

## Materials and methods

BALB/c and C57BL/6 mice, aged 6–8 weeks, were procured from Zydus Research Centre, Ahmedabad, India. All experimental procedures were reviewed and approved by the Institutional Animal Ethics Committee (IAEC) of Nirma University, Ahmedabad (protocol No.: IS/PHD/30/2022/46). The animal facility operates under the regulations of the Committee for Control and Supervision of Experiments on Animals (CPCSEA), Ministry of Fisheries, Animal Husbandry and Dairying, Government of India (Registration No. 883/PO/ReBi/S/05/CPCSEA).

Mice were acclimatized for 1 week in the Central Animal House Facility and provided with adequate nutrition, water, nesting materials, and routine veterinary monitoring. All handling procedures strictly followed institutional animal ethical guidelines. For primary immunogenicity experiments, a group of six to seven mice were used in accordance with the 3R principles Replacement, Reduction, and Refinement. Where appropriate, data from independent experiments were pooled to obtain sufficient statistical power. An initial/single adjuvant study was carried out using n=3–4 mice/group. C57BL/6 mice (IgG2c as a Th1 marker) were used for *Pb*SLTRiP and BALB/c mice (IgG2a as a Th1 marker) for *Pf*CSP immunizations. This strain selection leverages their complementary immune profiles and follows established practices in *Plasmodium* vaccine research. IgG subclass ELISAs specifically detected IgG2c (C57BL/6) and IgG2a (BALB/c) as Th1 markers, alongside total IgG and IgG1.

### Reagents

All the reagents used for the experimental work are cell culture tested: Poly(I:C) (LMW-25 mg) (InVivogen-tlrl-picw); R848 (Sigma SMLL0196–10 MG); 3,3′,5,5′-tetramethylbenzidine (TMB) Liquid Substrate System for ELISA (Sigma T0440–100 ML); carbonate–bicarbonate buffer (Sigma C3041-50CAP); bovine serum albumin (BSA) fraction-V, cell culture tested (HiMedia: TC194–100 G); goat anti-mouse IgG (H+L) Secondary Ab, HRP (Invitrogen 62-6520); goat anti-mouse IgG1 Secondary Ab, HRP (Invitrogen PA1-74421); goat anti-mouse IgG2c, Fc gamma-specific Ab, HRP (CST 56970); goat anti-mouse IgG2a, Fc gamma-specific Ab, HRP (CST 33416); 10× PBS (HiMedia ML023–500 ML); 10× PBS (endotoxin free) (HiMedia ML164–100 ML); RPMI-1640 (HiMedia AL199A-500 ML); antibiotic–antimycotic solution 100× liquid (HiMedia A002–20 ML); L-glutamine 200 mM solution (HiMedia TCL012–20 ML); HEPES-1 M Solution Cell culture tested (HiMedia TCL021); phosphate-buffer saline with 0.05% Tween 20, pH 7.4 (Sigma P3563-10PAK); ELISA Plate (Immunoplate Strip Single Well Genetix-38296 or 96-well, high binding, detachable (HiMedia EP2-5X10NO binding buffer: Tris-buffered saline pH 7.5 or 8.0); elution buffer: 50 mM Tris–HCl, 15 mM reduced glutathione, pH 8.0; lysis buffer: 100 mM Tris, 250 mM NaCl, 10% glycerol, 0.5 mM EDTA, 0.05% Triton X-100, pH 8.8 with 0.02 mg/mL lysozyme (Bio Basic Inc.); and 1× Protease Inhibitor mixture (S8820-Sigma, SIGMAFAST Protease Inhibitor Tablets) (generally used to add 1 mL of stock in 100 mL of lysis buffer). For the stock solution of 1× protease inhibitor mixture, one tablet makes 100 mL of cocktail. One tablet is recommended for the inhibition of proteases present in a maximum of 20 g of cell extract.

### *P. falciparum* CSP

**Figure 1:**
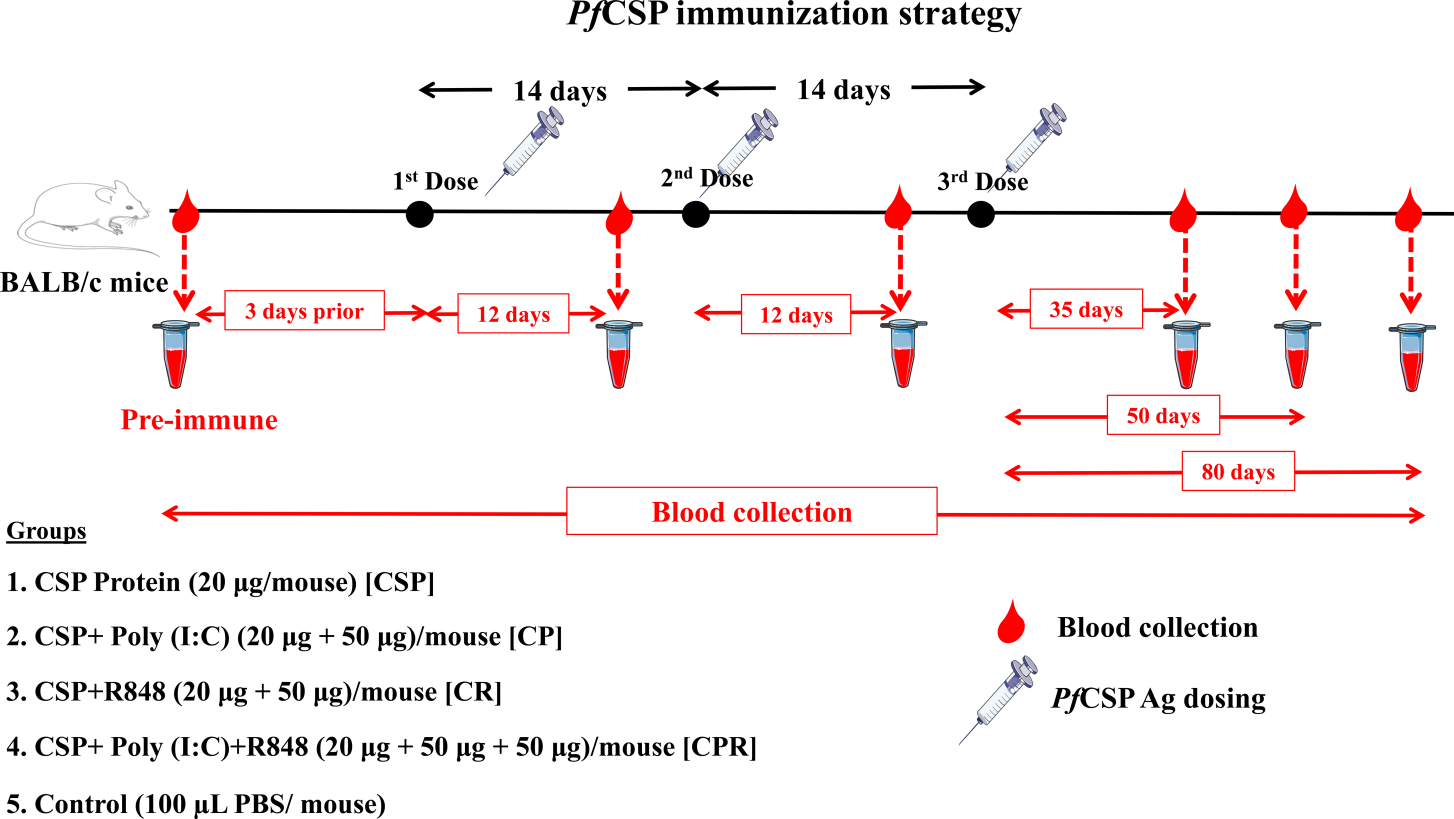
An outline for the immunization strategy for *P. falciparum* circumsporozoite protein (*Pf*CSP). This study outlines an immunization protocol for *Pf*CSP in BALB/c mice, including a timeline for antigen administration and blood sampling. Mice were immunized with three doses of the *Pf*CSP antigen, administered at 14-day intervals. Blood samples are collected at predefined time points to assess the mounting of the immune response. A baseline blood sample obtained 3 days prior to the initial immunization to determine preexisting immunity. Subsequent blood collections are performed 12 days following the first and second doses, as well as on days 35, 50, and 80 following the last immunization, to assess both intermediate and long-term immune responses. The experimental design includes five distinct groups: (1) *Pf*CSP antigen alone, (2) *Pf*CSP in combination with the adjuvant Poly(I:C) [CP], (3) *Pf*CSP in combination with the adjuvant R848 [CR], (4) *Pf*CSP co-administered with both Poly(I:C) and R848 [CPR], and (5) control group receiving phosphate-buffered saline (PBS).

The lyophilized (powder form) full-length recombinant *P. falciparum CSP* (*Pf*CSP) protein used for immunization (in BALB/c) was procured from Geneva Pharmaceuticals Ltd., Pune, India. The immunization procedure was similar to that described in our previous study (23), and the antigen details are similar to those reported in a previously published *Pf*CSP study (25). This immunogen was derived from the Indian strain IND637HDD1 (GenBank: AAN87606.1) of *P. falciparum*, retaining the murine H-2K^k^ cytotoxic T-cell epitope (_373_DYENDIEKKI_383_), conserved across major reference strains, including 7G8 and T4. The *Pf*CSP construct shares over 90% sequence identity with standard reference strains (3D7 and 7G8), and its use in preclinical vaccine studies has been previously documented (26). Lyophilized vials were reconstituted in NF-grade water following manufacturer instructions prior to use. Endotoxin levels were below the acceptable range (<10 EU/mL) for *Pf*CSP antigen batches. The immunization schedule is shown in **Figure 1**.

### *P. berghei* sporozoite- and liver stage-expressed tryptophan-rich protein

The SLTRiP protein, characterized by high tryptophan content and containing putative B- and T-cell dominant epitopes, is a conserved protein and promising candidate antigen for malaria LS infection studies. The DNA construct encoding *Pb*SLTRiP was kindly provided by Dr. Agam P. Singh (National Institute of Immunology (NII), New Delhi). The recombinant *Pb*SLTRiP protein was expressed and purified using affinity chromatography with minor modifications to the published protocols. Details of the modified purification method are provided in supplementary information (SI). Purity of the final protein preparation was verified by SDS-PAGE. The purified *Pb*SLTRiP protein was subsequently used for immunization in C57BL/6 mice according to the established protocol (23, 27). Endotoxins from the antigen preparation were removed using an endotoxin removal kit (Thermo-Pierce, USA). Endotoxin levels were tested in another protein batch using an endotoxin quantitation kit (Thermo-Pierce, USA). Endotoxin levels were below the acceptable range (≤10 EU/mL). Furthermore, we used GST alone in our experiments. For the purification of GST, the vector pGEX6P1 was transformed into *E coli*, cultured, and induced with IPTG in a similar manner to the SLTRiP-GST vector. GST protein was purified on a 5.0-mL prepacked GST-FF column (Cytiva) in a similar manner used for *Pb*SLTRiP mentioned in SI. Furthermore, preliminary ELISA confirmed no cross-reactivity of *Pb*SLTRiP-immunized sera with GST alone.

### Immunization schedule

BALB/c mice were selected for *Pf*CSP immunization based on an extensive precedent in CSP vaccine literature, where the H-2K^d^-restricted CD8^+^ T-cell epitope NYDNAGTNL is recognized, facilitating hypothesis testing aligned with established immunological methods. C57BL/6 mice were used for *Pb*SLTRiP, as per two published studies characterizing this LS antigen, ensuring consistency. BALB/c (IgG2a Th1 marker) for humoral-focused secreted *Pf*CSP and C57BL/6 (IgG2c Th1 marker) for cellular-focused intracellular *Pb*SLTRiP optimizes readouts matching antigen biology. Mice received three subcutaneous (SQ) immunizations at the base of the tail with LS malaria protein antigens, following our previously published protocol (23). Briefly, *Pf*CSP and *Pb*SLTRiP Ag(s) were administered either alone or formulated with adjuvants poly(I:C) and R848 to BALB/c and C57BL/6 mice at 2-week intervals for three doses, respectively. Blood samples were collected from the retro-orbital plexus puncture (under general anesthesia) of the mice using a sterile hematocrit capillary tube before (pre-immune, 2–3 days prior) and after each antigen/protein booster immunization, ideally 2 days preceding each booster dose, as outlined in **Figure 1** (for *Pf*CSP) and **Supplementary Figure S3B** (for *Pb*SLTRiP). After collection, blood was allowed to clot at 37°C for 3–4 h and then centrifuged at 1,000 ×g for 20 min at 4°C. The resulting serum was transferred to fresh microcentrifuge tubes and stored at −80°C until used.

### *Pf*CSP and *Pb*SLTRiP- specific IgG and IgG subclass ELISA

Antigen-specific total IgG, IgG1, IgG2c/IgG2a, and IgM in serum were quantified by indirect ELISA, performed as described previously (23). Briefly, 96-well immuno-plates were coated overnight at 4°C with *Pf*CSP (1 µg/well) and *Pb*SLTRiP (0.5 µg/well) diluted in coating buffer. The plates were then blocked with 2% BSA in PBS (200 μL/well) for 2 h at room temperature (RT). Serially diluted serum samples (1:500 to 1:512,000; 100 μL/well) were added and incubated for 2 h at RT. After three washes with PBS containing 0.05% Tween 20 (PBST; 200 μL/well), HRP-conjugated secondary antibodies were added (anti-mouse IgG (H+L), 1:2,000; anti-mouse IgG1, 1:4,000; anti-mouse IgG2c, 1:1,000, anti-mouse IgG2a, 1:3,000) (100 μL/well)) and incubated for 1 h at RT. The plates were washed again with PBST followed by addition of the TMB substrate (100 μL/well). The reaction was stopped with 1 M HCl (100 μL/well), and optical density (OD) was measured at 450 nm with reference at 620 nm using a microplate reader (Microplate Spectrophotometer, Epoch SN). ELISA was performed in duplicate wells for each serum sample.

### Antibody endpoint titer determination

*Pf*CSP-specific IgG endpoint titers were determined from ELISA dilution curves using a statistically defined cutoff. For each plate, pre-immune sera from the corresponding immunization group were tested at the same serial dilutions, and for every dilution, the mean OD_450_ and standard deviation (SD) of pre-immune samples were calculated. The cutoff for positivity at each dilution was defined as mean_pre-immune_+ 3SD_pre-immune_, which corresponds to a signal clearly above background noise and is widely used for endpoint-titer ELISAs. For each mouse and time point, the endpoint titer was defined as the reciprocal of the highest serum dilution at which the OD_450_ remained above the corresponding cutoff. Endpoint titers were plotted on a logarithmic (log_10_) scale for graphical representation.

### Avidity index

The avidity index (AI) of IgG antibodies was assessed to evaluate the strength of antigen–antibody interactions, following a modification of the standard ELISA protocol (23). In summary, after the primary serum incubation step, half the wells of each plate were treated with 6 M urea for 10 min to disrupt low-affinity binding, while the remaining control wells received 1× PBS. Serum dilutions were predetermined to ensure uniform OD values across groups. Additionally, the AI was also computed for each immunization dose/group, utilizing the entire range of dilutions for each dose. The AI, computed as the ratio of mean OD values from urea-treated wells (A_6M urea_) to PBS-treated control wells (A_c_), was determined. Multiple serum dilutions were employed, and all samples were assessed in duplicate.

### Sporozoite isolation and development of challenge model to assess the vaccine potential of LS antigens

*Anopheles stephensi* mosquitoes and *Plasmodium berghei* (ANKA) parasites were provided by Dr. Agam Prasad Singh, NII, New Delhi, India. The cultivation of the vector and parasite was conducted at the vivarium facility of Institute of Science, Nirma University, Ahmedabad, India, strictly adhering to the Institutional Biosafety Committee (IBSC) regulations. Mosquitoes were reared at 25°C–27°C and at 65%-70% humidity. For infection cycles and biting, the temperature was precisely controlled at 19 °C–21 °C and humidity at 90%-95%, maintained via automated sensor technologies under a 12-h light/dark cycle. During the biting phase, feeding also lasted 7 to 8 min, but the mosquitoes needed to be disturbed by gentle tapping, and mouse position was changed every 30 s to encourage biting. Uninfected mosquitoes (2 to 6 days old) were blood-fed on mice infected with *P. berghei* (ANKA) displaying 0.5% to 1.0% gametocyte levels. After 18–21 days postinfection, sporozoites (SPZs) were obtained by dissecting the salivary glands, following the established protocol (10, 28).

Furthermore, considering the findings of Jaijyan et al. (27), we utilized the established Infection/Cure model (29) to investigate the temporal expression dynamics of the recombinant *Pb*SLTRiP protein during *P. berghei* SPZ infection. Experimental animals received two or three intravenous inoculations of *P. berghei* SPZs (10,000/20,000 per mouse, in 100 μL PBS), administered at 10-day intervals. Concurrently, starting on the day of the initial SPZ inoculation and continuing daily for 9 days following the final inoculation, the animals were treated with chloroquine (CQ) (800 μg/mouse in 100 μL PBS) via intraperitoneal (i.p.) injection. This CQ administration prevented the development of lethal blood-stage parasitemia, thereby facilitating prolonged exposure to LS antigens and the subsequent development of durable immune memory. The control group included animals receiving identical CQ treatment without SPZ inoculation (CQ control) and an absolute PBS control group. Serum samples were collected at different intervals postinfection to monitor temporal *Pb*SLTRiP expression dynamics via ELISA.

### *In silico* analysis of adjuvant–receptor interactions

To elucidate the molecular basis of dual adjuvant synergy, structural visualization of TLR3–poly(I:C) (PDB 7WV5) and TLR8–R848 (PDB 3W3M) interactions was performed using BIOVIA Discovery Studio Visualizer. Hydrogen bonds, salt bridges, polar contacts, hydrophobic interactions, and π–π stacking between receptor chains and ligands were identified using the interaction analysis tools of the software. Homology models of murine TLR7 and TLR8 ectodomains were generated using SWISS-MODEL with corresponding human structures (TLR7: PDB 6LVX; TLR8: PDB 3W3M) as templates. Mouse TLR7 (UniProt P58681) and TLR8 (UniProt P58682) ectodomain sequences (approximately residues 27–830 for TLR7 and 28–860 for TLR8) were submitted in automated mode. Top-ranking models were selected based on Global Model Quality Estimation (GMQE) and QMEAN scores, confirming preservation of the characteristic LRR horseshoe architecture and ligand-binding pocket conservation relative to human templates.

### Statistical analysis

Prior to statistical analyses, all continuous variables were assessed for normal distribution using the Shapiro–Wilk test (or Kolmogorov–Smirnov test for larger sample sizes). Data were considered normally distributed if the *p*-value was greater than the predetermined significance level of 0.05. Data are shown as mean ± SEM. Although the distribution of endpoint titers did not significantly deviate from normality (p>0.05), non-parametric tests were applied because of the small sample size (n=3–4 mice/group). Differences in titers across time points within each immunization group were analyzed using the Kruskal–Wallis test followed by Dunn’s *post-hoc* multiple-comparison procedure, which does not rely on normality assumptions and is recommended for small-sample immunological titer data (GraphPad Software Inc., San Diego, CA, USA). To compare endpoint titers between CSP and SLTRiP immunization groups at each dose, Mann–Whitney U tests were performed. Pairwise comparisons were conducted separately at each time point (among groups) without adjustment for multiple comparisons. The statistical significance thresholds are denoted as: ns (not significant), *p < 0.05, **p < 0.01, ***p < 0.001, ****p < 0.0001.

## Results

### Repeated *Pf*CSP immunization maintains long-lasting IgG endpoint titers

**Figure 2:**
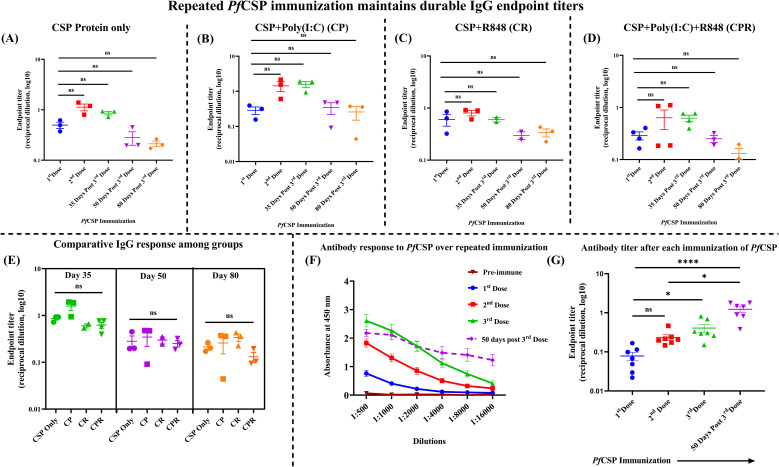
Repeated *Pf*CSP immunization maintains durable IgG endpoint titers with different adjuvant formulations. **(A–D)***Pf*CSP-specific total IgG endpoint titers in mice immunized with **(A)** CSP protein only, **(B)** CSP+Poly(I:C) (CP), **(C)** CSP+R848 (CR), or **(D)** CSP+Poly(I:C)+R848 (CPR). Sera were collected pre-immune, after the first and second doses, and at 35, 50, and 80 days following the third dose of antigen administration. Each symbol represents an individual mouse; horizontal bars indicate median reciprocal endpoint titers (cutoff defined at OD_450_ above pre-immune baseline). **(E)** Cross-group comparison of *Pf*CSP-specific IgG endpoint titers at 35, 50, and 80 days post-third dose for CSP, CP, CR, and CPR regimens. **(F)***Pf*CSP-specific IgG ELISA titration curves for the expanded CPR cohort, showing mean OD_450_ values ± SEM across serum dilutions (1:500–1:16,000) at pre-immune, first, second, and third doses and 50 days post third dose of antigen administration **(G)***Pf*CSP-specific IgG endpoint titers for the expanded CPR cohort at each immunization and at 50 days post third dose. Symbols represent individual mice with median values, illustrating the progressive increase in titers and their maintenance at the latest time point. The data represented here is the pool of two independent experiment (n = 6–7 mice/group for main immunization; n=3–4 mice/group for single adjuvant and initial pilot studies). Data are presented as mean ± SEM. For all the Y-axis **(A–E, G)**, it is represented as the following: Endpoint titers (log_10_ reciprocal dilution). Differences in titers across time points within each immunization group were analyzed using the Kruskal–Wallis test followed by Dunn’s *post-hoc* multiple-comparison procedure. To compare endpoint titers between CSP immunization groups at each dose, non-parametric Mann–Whitney U tests were performed. Pairwise comparisons between groups were conducted separately at each time point without adjustment for multiple comparisons, with significance denoted as ns (not significant), *p < 0.05, **p < 0.01, ***p < 0.001, ****p < 0.0001.

In an initial cohort, *Pf*CSP-specific total IgG responses were evaluated after repeated immunization with CSP alone or in combination with Poly(I:C) (CP), R848 (CR), or dual Poly(I:C)+R848 (CPR) adjuvants. Serial dilution ELISA curves for each group are shown in **Supplementary Figure S1**, and the corresponding endpoint titers are summarized in **Figures 2A–D**. Across all regimens, endpoint titers increased after the second and third doses and then declined at later time points, consistent with expansion and contraction of the antibody response. All groups reached peak titers of approximately log_10_ 4.2 (at the dilution of 1:16,000) by 35 days post-third dose. Thereafter, endpoint titers decreased in every group: CP showed the slowest decline, while CSP, CR, and CPR exhibited lower titers on days 50 and 80 post-third dose. A comparative analysis of endpoint titers on days 35, 50, and 80 (**Figure 2E**) did not reveal statistically significant differences among CSP, CP, CR, and CPR, indicating that none of the adjuvant regimens achieved clear statistical superiority over the others at these time points.

### Extended analysis of the CPR regimen in a larger cohort

Based on the current trends seen in the immune responses and published work using the same dual-adjuvant combination with OVA, which showed that this dual TLR3/TLR7/8 agonist system can enhance antigen-specific B- and T-cell responses, an expanded cohort was immunized with the CPR formulation (30, 31). ELISA dilution curves for the larger CPR group (**Figure 2F**) and the corresponding endpoint titers at each dose and at 50 days post-third dose are shown (**Figure 2G**). In this cohort, *Pf*CSP-specific IgG endpoint titers increased with each immunization and remained high 50 days after the third dose. At this time point (**Figure 2G**), CPR titers were in the same range as those observed for CP (**Figure 2B**) and higher than those with CR (**Figure 2C**). However, these differences did not reach statistical significance.

### Poly(I:C)+R848 is associated with altered IgG1 and IgG2c endpoint titers in *Pf*CSP-immunized mice

**Figure 3:**
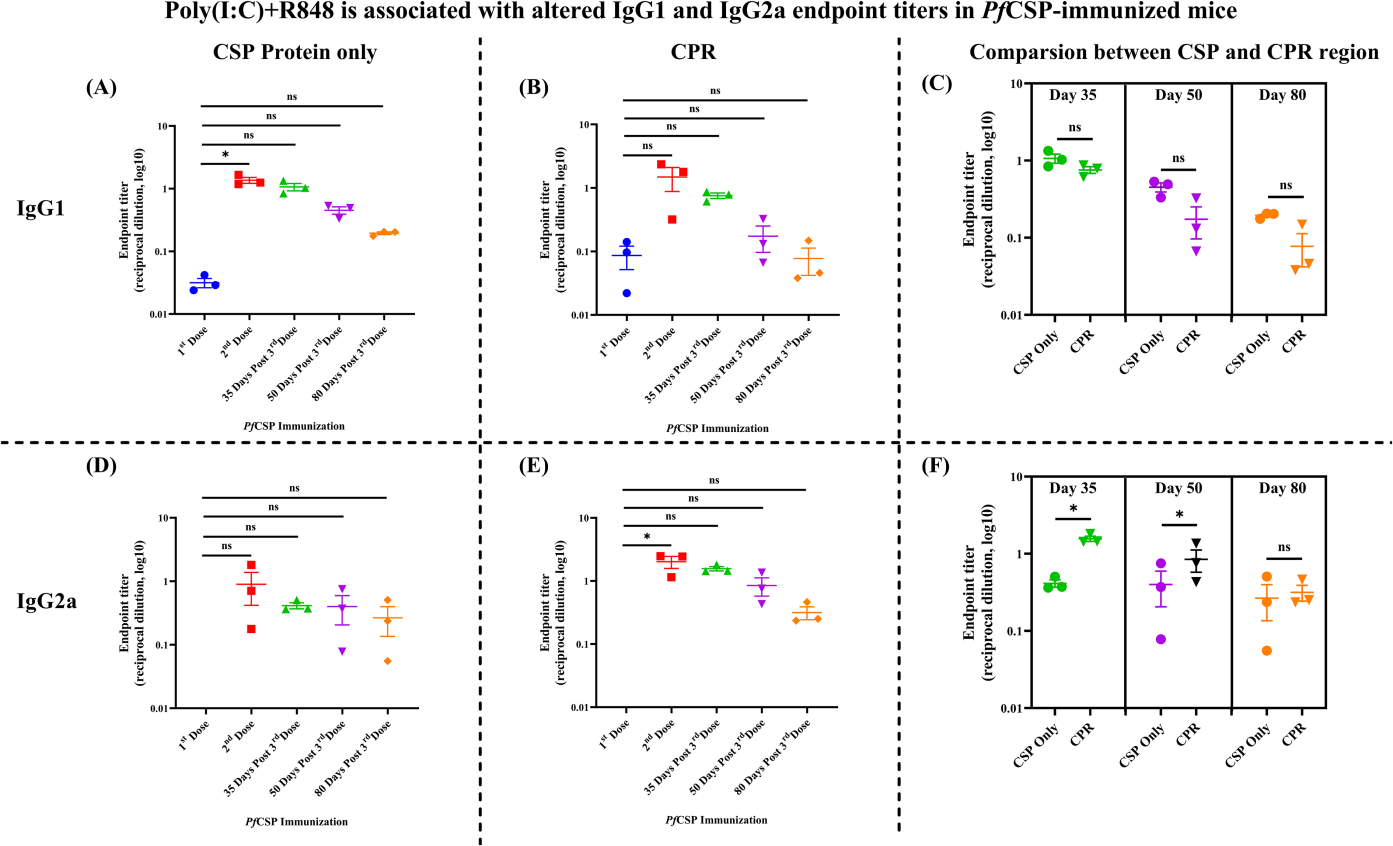
Poly(I:C)+R848 is associated with altered IgG1 and IgG2a endpoint titers in *Pf*CSP-immunized mice. **(A, B)***Pf*CSP-specific IgG1 endpoint titers in mice immunized with CSP protein only **(A)** or CSP+Poly(I:C)+R848 (CPR, **B**). Sera were collected after the first and second doses and at 35, 50, and 80 days post third dose of antigen administration. Each symbol represents an individual mouse; horizontal lines indicate median reciprocal endpoint titers (log_10_ scale). **(C)** Direct comparison of IgG1 endpoint titers between CSP-only and CPR groups at 35, 50, and 80 days post third dose of antigen administration. Data are shown as individual mice with median values; differences at each time point were assessed by Mann–Whitney test (ns). **(D, E)***Pf*CSP-specific IgG2a endpoint titers for the CSP-only **(D)** and CPR **(E)** groups at the same time points. Symbols and statistics as in panels **(A, B, F)** Direct comparison of IgG2a endpoint titers between the CSP-only and CPR groups at 35, 50, and 80 days post third dose of antigen administration. n=3–4 mice/group for single adjuvant and initial pilot studies. Data are presented as mean ± SEM. For all the Y-axis **(A–F)**, it is represented as the following: Endpoint titers (log_10_ reciprocal dilution). Differences in titers across time points within each immunization group were analyzed using the Kruskal–Wallis test followed by Dunn’s *post-hoc* multiple-comparison procedure. To compare endpoint titers between CSP-only and CPR immunization groups at each dose, non-parametric Mann–Whitney U tests were performed. Pairwise comparisons between groups were conducted separately at each time point without adjustment for multiple comparisons, with significance denoted as ns (not significant), *p < 0.05, **p < 0.01, ***p < 0.001, ****p < 0.0001.

To assess qualitative features of the antibody response, IgG1 and IgG2a subclasses were examined in CSP-only and CPR groups. The respective titration curves for both IgG1 and IgG2a are shown in **Supplementary Figure S2**, and the corresponding endpoint titers are summarized for IgG1 (**Figures 3A, B**) and IgG2a (**Figures 3D, E**). In both groups, IgG1 endpoint titers increased after booster doses and then declined between days 50 and 80, in line with total IgG kinetics. CSP alone produced robust IgG1 titers but lower IgG2a titers, whereas CPR immunization produced similar IgG1 titers, together with consistently detectable IgG2a across time points.

Comparisons between CSP and CPR at the follow-up time points are presented for IgG1 (**Figure 3C**) and IgG2a (**Figure 3F**). IgG1 endpoint titers did not differ significantly between CSP and CPR on days 35, 50, or 80, suggesting that the dual adjuvant did not substantially alter the magnitude of the IgG1 response at these later time points. In contrast, IgG2a endpoint titers were higher in the CPR group than in the CSP group on days 35 and 50, with statistically significant differences, whereas by day 80, the difference was no longer significant, and the median titers in both groups had declined.

### SLTRiP protein induces antibody responses augmented by Poly(I:C)+R848 (SPR)

**Figure 4:**
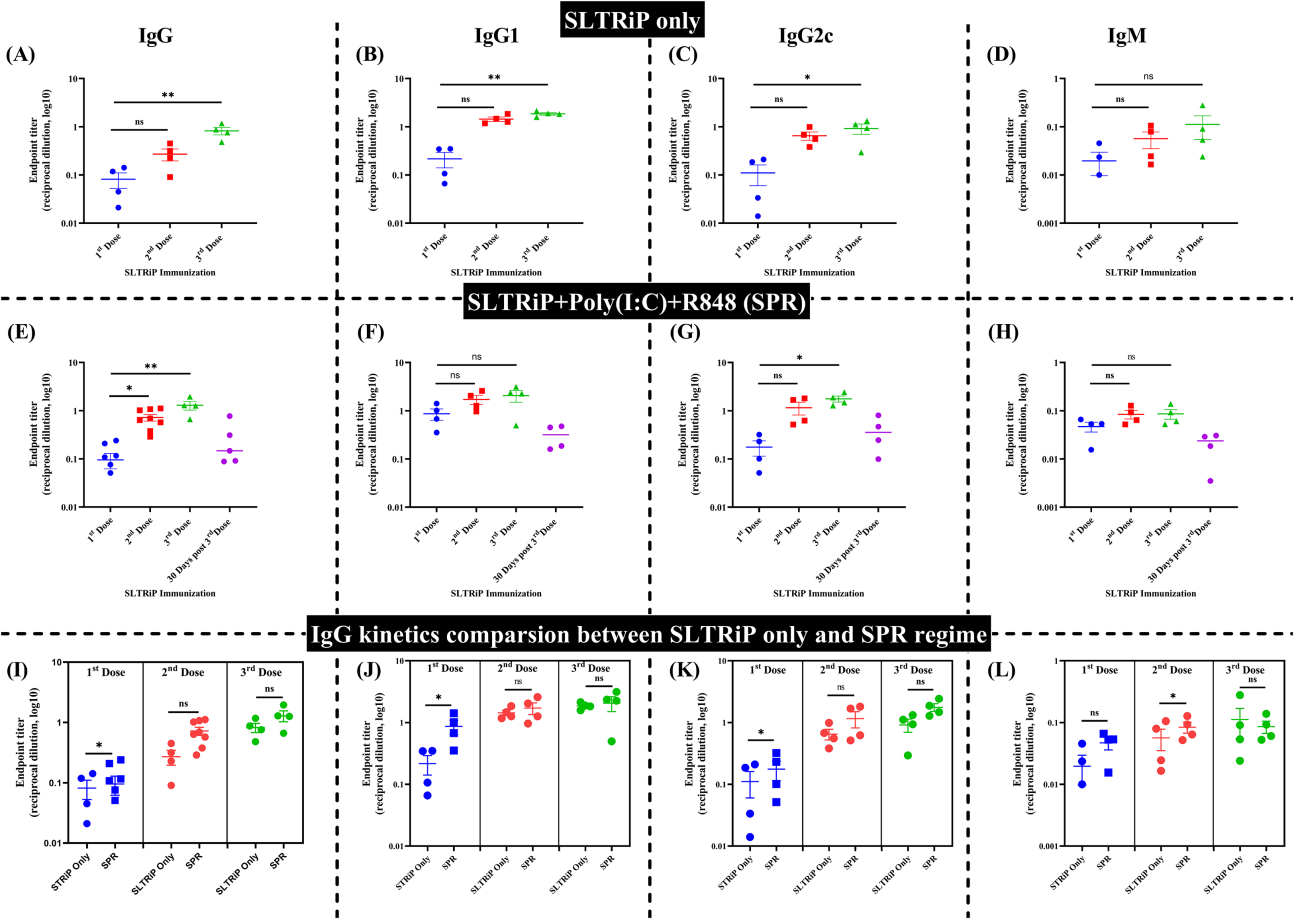
*Pb*SLTRiP-specific IgG, IgG subclass, and IgM endpoint titers with and without Poly(I:C)+R848 (SPR). **(A–D)** Endpoint titers (log_10_ reciprocal dilution) of total IgG **(A)**, IgG1 **(B)**, IgG2c **(C)**, and IgM **(D)** in mice immunized with *Pb*SLTRiP protein only. Sera were collected after the first and second doses and at the third dose; each symbol represents an individual mouse, and horizontal bars show median titers. **(E–H)** Corresponding endpoint titers for mice immunized with *Pb*SLTRiP formulated with Poly(I:C)+R848 (SPR), measured after the first, second, and third doses and at 30 days post third dose. Symbols and statistics as in panels **(A–D)**. **(I–L)** Direct comparison of endpoint titers between *Pb*SLTRiP-only and SPR groups for total IgG **(I)**, IgG1 **(J)**, IgG2c **(K)**, and IgM **(L)** at each dose. Differences in titers across time points within each immunization group were analyzed using the Kruskal–Wallis test followed by Dunn’s *post-hoc* multiple-comparison procedure. The *Pb*SLTRiP-only group contains n=3–4 mice/group, while the SPR-adjuvanted group represents the pooled data from two independent experiments (n=4 mice/group). Data are presented as mean ± SEM. For all the Y-axis **(A–F)**, it is represented as the following: Endpoint titers (log_10_ reciprocal dilution). To compare endpoint titers between CSP-only and CPR immunization groups at each dose, non-parametric Mann–Whitney U tests were performed. Pairwise comparisons between groups were conducted separately at each time point without adjustment for multiple comparisons, with significance denoted as ns (not significant), *p < 0.05, **p < 0.01, ***p < 0.001, ****p < 0.0001.

Following the *Pf*CSP observations, the immunogenicity of *P. berghei* SLTRiP (*Pb*SLTRiP), another recombinant LS protein, was evaluated. We expressed and purified the *Pb*SLTRiP protein (27), which showed the expected *Pb*SLTRiP-GST fusion (75 kDa) and *Pb*SLTRiP band (25 kDa) in the SDS-PAGE (**Supplementary Figure S3A**), and the immunization schedule is given in **Supplementary Figure S3B**. ELISA titration curves for *Pb*SLTRiP-only and SPR-immunized mice demonstrated progressive increases in total IgG, IgG1, IgG2c, and IgM across successive doses (**Supplementary Figures S3C–J**), with GST alone not inducing a measurable response (**Supplementary Figure S4**). Endpoint titers were calculated using a maximum dilution of 1:16,000, reflecting the limit observed in pre-immune sera (**Figures 4A–H**). In the *Pb*SLTRiP-only group, total IgG, IgG1, and IgG2c endpoint titers increased significantly between first and third immunizations (**Figures 4A–C**), whereas IgM titers showed an early peak followed by a plateau (**Figure 4D**). In the SPR group, total IgG and subclass endpoint titers were higher following the third dose and persisted for 30 days (**Figures 4E–G**). Moreover, IgM titers followed a similar pattern showing higher overall magnitudes (**Figure 4H**).

Direct comparisons between *Pb*SLTRiP-only and SPR regimens at each dose revealed differences associated with the dual TLR adjuvant on the magnitude and quality of the response (**Figures 4I–L**). After the first immunization, SPR mice showed significantly higher endpoint titers than the *Pb*SLTRiP-only group for total IgG, IgG2c, and IgM, while IgG1 showed a similar trend. By the second and third doses, endpoint titers in both groups had converged, and no consistent statistically significant differences were detected, although SPR showed to maintain numerically higher total IgG and IgG2c titers at the latest point. Overall, these observations suggest that *Pb*SLTRiP mounted the heightened antibody response, whereas the dual adjuvants, associated with earlier responses, did not show statistically significant differences at later time points.

### *Pb*SLTRiP-specific antibody avidity under SLTRiP-only and SPR regimens

The functional aspects of *Pb*SLTRiP-specific antibodies were further explored by measuring antibody avidity using urea-displacement ELISA. Avidity index (AI) values increased moderately over successive immunizations in the *Pb*SLTRiP-only group. These alterations, however, did not reach statistical significance (**Supplementary Figure S5A**), consistent with the relatively small shift observed in the corresponding urea-ELISA curves (**Supplementary Figure S5B**). In the SPR regimen, AI values and urea-ELISA curves were consistent with higher avidity after the third immunization, with values at 30 days post-third dose remaining similar to those seen at earlier time points (**Supplementary Figures S5C, D**). This paves the way for exploring dual-adjuvant effects on SLTRiP-specific B-cell and T-cell responses to liver-stage malaria antigens.

**Figure 5:**
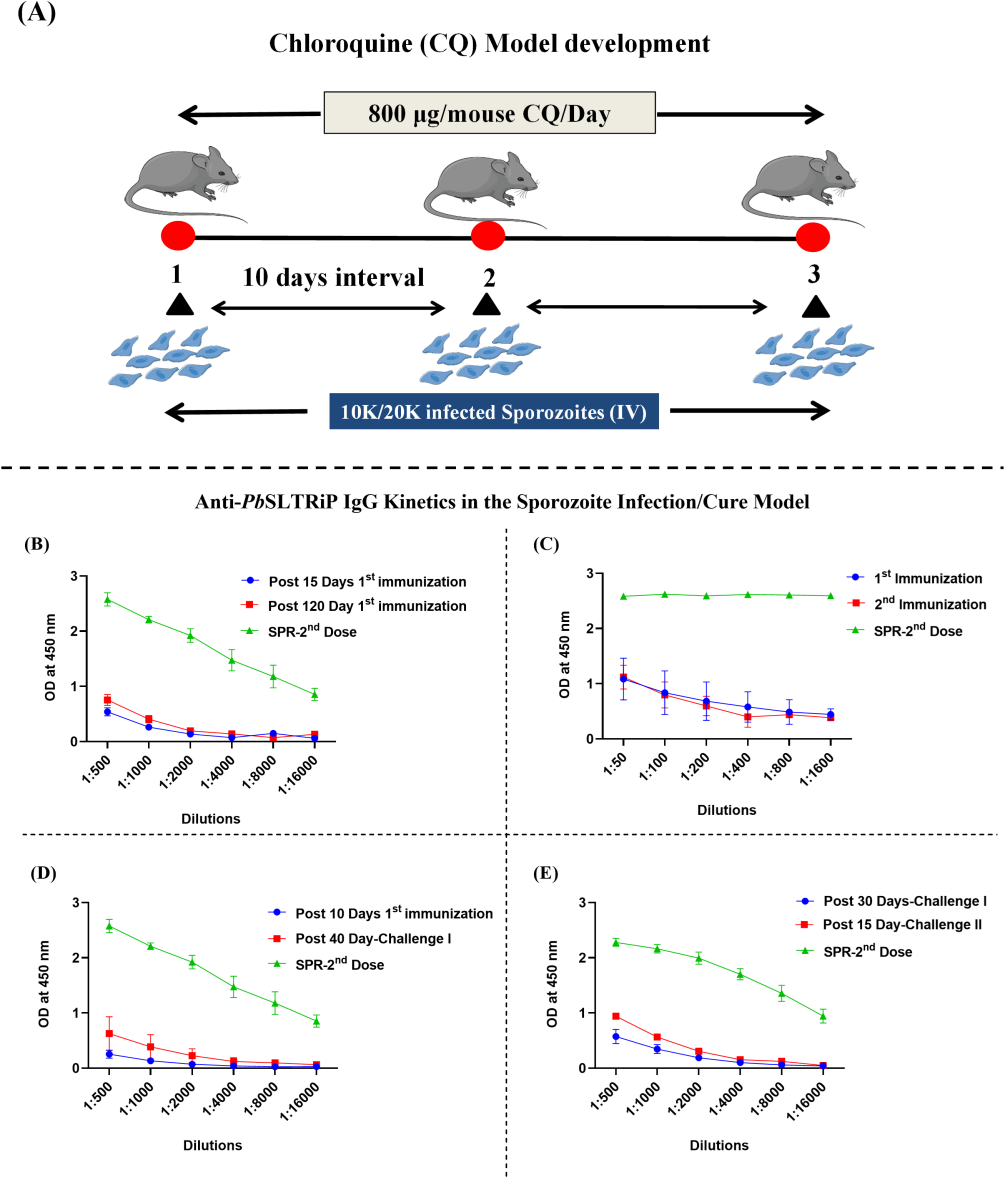
*Pb*SLTRiP antigen expression dynamics are determined by the chemoprophylaxis and sporozoites (CPS) model. **(A)** Schematic presentation of the CPS model. Mice received intravenous (IV) inoculation of 10,000-20,000 *P. berghei* SPZs concurrently with daily intraperitoneal (i.p.) CQ treatment (800 µg/mouse/day) for sequential doses (1,2,3), separated by a 1-day interval. **(B)***Pb*SLTRiP-specific IgG kinetics following the first SPZ inoculation. ELISA titration curves show the modest humoral response measured 15 days and 120 days following the first inoculation under CQ pressure. **(C)***Pb*SLTRiP-specific IgG response following sequential inoculations. ELISA titration curves comparing the anti-*Pb*SLTRiP IgG level measure after the first and second SPZ inoculations, demonstrating enhancement upon repeated antigen exposure in the CPS setting. *Pb*SLTRiP-specific IgG response **(D)** after initial challenges by observing the antibody response 40 days post-challenge I **(E)** after subsequent challenge in which antibody response was measured 30 days following the first challenge and 15 days following the second challenge. OD measured at 450 nm vs. serum dilutions are shown. Positive control represents serum from the immunized SPR group after the second dose. n=3–5 mice/group/experiment.

To validate the translational relevance of *Pb*SLTRiP, the immune response against this protein was assessed during pre-erythrocytic infection using the chemoprophylaxis and sporozoites infection (CPS)/cure model/CQ model) (**Figure 5A**). Mice received repeated SPZ inoculations under CQ cover, and *Pb*SLTRiP-specific IgG responses were monitored over time, with serum from SPR-immunized (following the second dose) group included as a positive control (**Figures 5B, E**).

Following the first sporozoite inoculation with CQ treatment, a modest but detectable anti-*Pb*SLTRiP antibody response was observed on days 15 and 120 post-inoculation (**Figure 5B**). This finding is consistent with *Pb*SLTRiP immunogenicity during early LS infection when blood-stage development is suppressed. Since *Pb*SLTRiP is expressed in both sporozoites and LS parasites (27), the observed response may reflect combined antigen exposure from these stages. A second sporozoite inoculation led to a clear increase in anti-*Pb*SLTRiP IgG levels compared to that with first exposure (**Figure 5C**). This indicated the cumulative antigen encounters as well as improved priming.

Subsequently, the effect of an unsuppressed challenge infection was examined. In mice that had completed the CPS regimen, a higher-dose SPZ challenge induced a marked increase in anti-*Pb*SLTRiP antibody titers by day 40 post-challenge (**Figure 5D**), which was consistent with the recall response. Elevated anti-*Pb*SLTRiP IgG levels were maintained following both the first and second challenge infections (**Figure 5E**) consistent with infection-induced priming supporting durable humoral memory against this LS antigen. These infection-model data, together with the vaccination results, are consistent with dual TLR-based adjuvant combinations, providing a rationale for exploring B-cell-and T-cell responses to *Pb*SLTRiP and other LS malaria vaccine antigens.

### *In silico* epitope mapping of LS antigens (*Pf*CSP and *Pb*SLTRiP)

**Figure 6:**
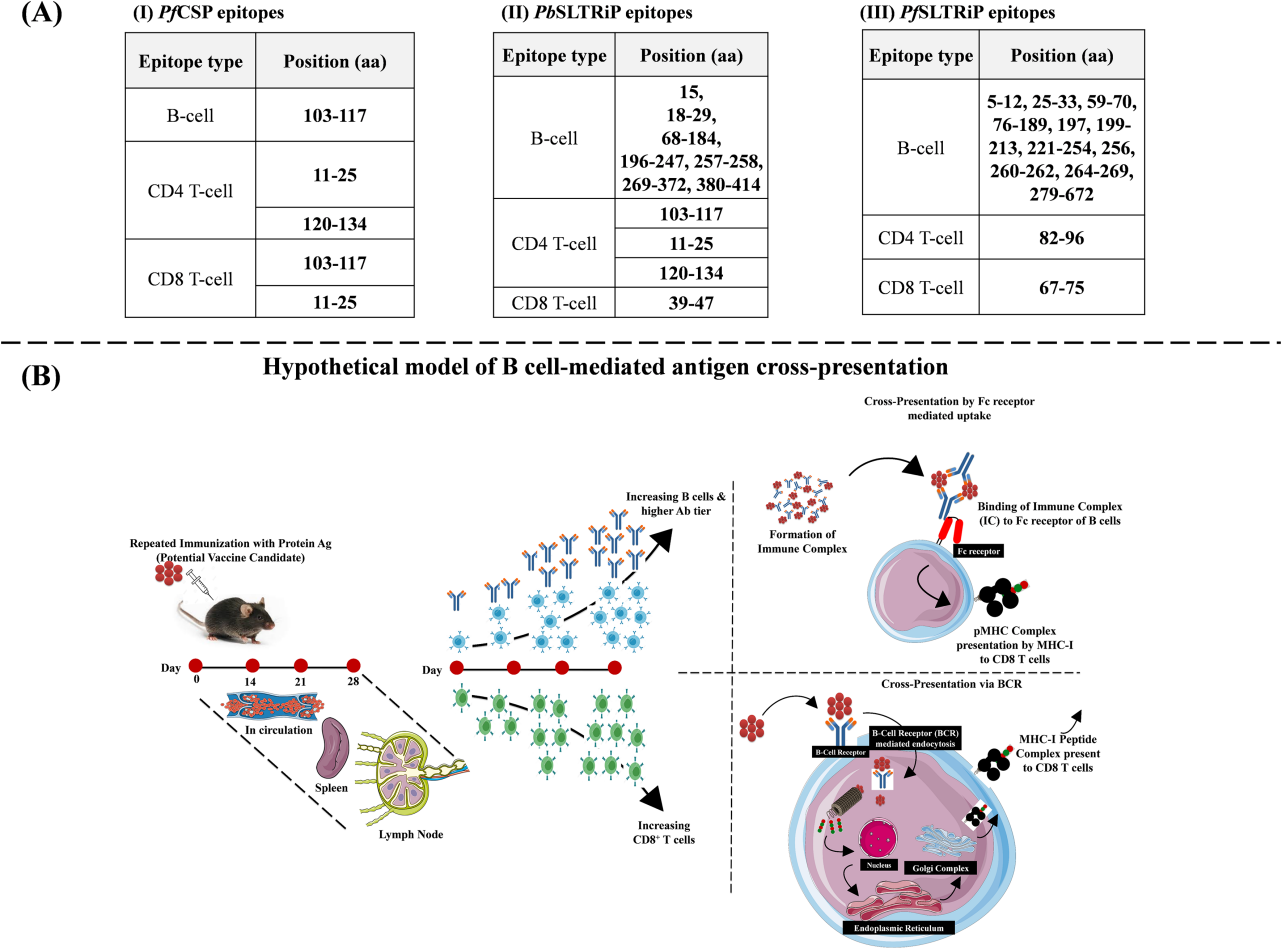
**(A)***In silico* identification of B-cell, CD8^+^, and CD4^+^ T-cell epitope using IEDB for CSP and SLTRiP malaria liver-stage protein antigens. With the usage of BepiPred Linear Epitope Prediction 2.0, NetMHCpan 4.1 EL, and NetMHCIIpan 4.1 EL of the IEDB tool, epitopes were identified for **(A-I)** CSP of *P. falciparum*, and SLTRiP of **(A-II)***P. berghei, and***(A-III)***P. falciparum*. The parameters were set to the default throughout the epitope identification. Furthermore, H-2K^b^ or H-2D^b^ and H-2K^d^ or H-2D^d^ have been used (peptide length=8/9 mer) for CD8^+^ T-cell epitope identification in C57BL/6 (for SLTRiP) and BALB/c (for CSP) mice, respectively. **(B)** Proposed mechanism for B-cell-mediated cross-presentation of protein Ag(s) to activate CD8^+^ T cells. Repeated immunization with protein antigens can effectively induce both humoral (characterized by the production of antigen-specific IgG antibodies) and cell-mediated (activation of CD4^+^ and CD8^+^ T cells) immune responses. Repeated immunizations with protein antigens or whole sporozoite vaccine (WSV) that promote humoral responses are expected to generate and expand memory B cells, with the affinity of their B-cell receptors (BCRs) likely to improve with each protein/subunit vaccine dosage. Possibly for antigen-specific B cells produced before a booster dose, vaccine administration would bind to the antigen, containing both B- and T-cell epitopes, leading to antibody secretion and the presentation of the antigen to the CD8^+^ T cells via cross-presentation. The high-titer and high-affinity antibodies produced over multiple immunizations may form immune complexes (ICs) with freely circulating antigen or incoming antigen during subsequent vaccine doses. These ICs could be captured by antigen-presenting cells (APCs), such as dendritic cells (DCs) and macrophages (Mϕs), through Fc receptors (FcRs), processed, and then presented to CD8^+^ T cells.

To provide a computational framework for the observed antibody responses and subclass profiles, we performed *in silico* epitope mapping for *Pf*CSP and *Pb*SLTRiP. Using the Immune Epitope Database (IEDB) analysis resource, we identified several linear predicted B-cell epitope clusters in both antigens (32), which aligned with the detectable antibody response measured experimentally (**Figure 6A**; **Supplementary Figure S6**). High-affinity predicted CD8^+^T cell epitopes were identified using NetMHCpan 4.1 EL (33), including CS_39–47_ within *Pf*CSP (34, 35) and SLTRiP_39-47_ (for *P. berghei*) (36) and SLTRiP_67-75_ (for *P. falciparum*), and associated with the cytotoxic T-cell activation.

CD4^+^ T-cell epitope prediction using NetMHCIIpan 4.1 EL predicted that many top-scoring MHC II binding regions overlap with these putative B-cell epitope clusters (**Figure 6A**). Together with favorable VaxiJen (https://www.ddg-pharmfac.net/vaxijen/VaxiJen/VaxiJen.html) antigenicity scores for *Pb*SLTRiP (0.7653) and *Pf*SLTRiP (0.7132), these computational analyses suggest the regions potentially compatible with the engagement of both humoral and cellular arms of adaptive immunity requiring experimental validation, however.

Based on these epitope predictions and the IgG1 and IgG2 subclass responses observed with the dual-adjuvant formulation (CPR and SPR), we propose a hypothetical model suggesting that B cells may contribute not only to antibodies but also to antigen presentation (**Figure 6B**). In this conceptual framework, B cells may capture antigen–antibody complexes via Fc receptors or B-cell receptors, internalize them, and potentially cross-present processed peptides on MHC I and MHC II to CD8^+^ and CD4 T^+^ cells, respectively. This proposed B-cell-mediated cross-presentation mechanism needs an extensive functional experimental validation with LS antigens (*Pf*CSP and SLTRiP).

### *In silico* structural analysis supporting adjuvant synergy hypothesis

Structural inspection of the TLR3–poly(I:C) complex (PDB 7WV5) showed that dsRNA (chains E/F, blue/black) engages a positively charged groove formed by the TLR3 ectodomain dimer (chains A/B, red/yellow), with multiple Lys and Arg residues forming hydrogen bonds (green) and salt bridges (red) to the RNA phosphate backbone (**Supplementary Figure S7A**). These electrostatic interactions suggest strong TRIF-dependent sensing of Poly(I:C) by endosomal TLR3. Furthermore, structural inspection of the TLR8–R848 complex (PDB 3W3M) revealed R848 binding in a hydrophobic pocket formed by conserved LRRs, with hydrogen bonds from histidine/aspartate residues to the adenine moiety and π–π stacking interactions with aromatic side chains stabilizing the pose (**Supplementary Figure S7B**). These interactions are consistent with potent MyD88-dependent signaling by endosomal TLR7/8, complementarity with TLR3 activation by Poly(I:C) providing a structural rationale for exploring adjuvant synergy.

### Molecular conservation supporting murine translation of dual adjuvant design

To examine the applicability of human TLR structures to mouse models, homology models of murine TLR7 (UniProt P58681) and TLR8 (P58682) ectodomains were generated using SWISS-MODEL with human templates PDB 6LVX (TLR7) and 3W3M (TLR8), respectively (GMQE >0.7; QMEAN z-scores −1.2 to 0.5). The structural superposition revealed high conservation, human TLR7 (6LVX, gray) aligned with the murine TLR7 model (blue; Panel C) at RMSD of 0.8 Å across the LRR horseshoe (residues ~27–830), maintaining the central concave ligand-binding groove important for ssRNA/small agonist sensing. Human TLR8–R848 (3W3M, green; Panels A/B) superimposed on the murine TLR8 model (yellow; Panel D) at RMSD of 1.0 Å (residues ~28–860), conserving the hydrophobic pocket (Phe/Tyr/Leu residues), His373/Asp345 hydrogen bonds to R848 adenine, and π–π stacking with Trp648, important for MyD88 recruitment.

Human TLR3–poly(I:C) (PDB 7WV5; panels not shown) exhibits >95% groove conservation with mouse TLR3 (PDB 3CIG), whereas human TLR8–R848 (3W3M; Panel B) displays the physiological dimer conformation mirrored in the murine model. This ectodomain conservation (>80% sequence identity) suggests a basis for potent R848 responses in mice and is consistent with Poly(I:C)/R848 synergy through conserved TRIF/MyD88 pathway activation, which was observed in our immunizations (**Supplementary Figure 8**).

## Discussion

The quest for an effective pre-erythrocytic LS stage malaria vaccine needs long-lasting humoral and cellular immunity. Thus, we investigated whether a dual TLR agonist adjuvant formulation (combination of Poly(I:C) (TLR3) and R848 (TLR7/8)) increases immunogenicity of *Pf*CSP (25) and *Pb*SLTRiP (36, 37) antigens. Across both antigens, adjuvanted regimens showed trends toward slower titer decay relative to the unadjuvanted controls, and that too without statistical differences from single adjuvants (**Figures 2**–**4**).

No statistically significant differences were observed among small-cohort regimens (**Figures 2A–D**) at matched time points (Kruskal–Wallis test, p>0.05 across all comparisons). Similarly, the expanded CPR cohort (**Figures 2F, G**) showed no significant differences from the pilot CPR cohort at equivalent time points (day 50 post-dose 3; p>0.05), with endpoint titers falling within the range observed for the CP formulation in the pilot study. This reproducibility between pilot and expanded cohorts is consistent with biological consistency across experiments without demonstrating the superiority of dual adjuvants in augmenting the immune response. These observations show the statistical limitations of small sample sizes (n=3-4/group) but support the consistency in the trends observed. For *Pf*CSP, all adjuvanted formulations (CP, CR, and CPR) sustained titers beyond day 50 post third-dose administration as compared to CSP alone; CP showed the highest levels on day 80 (**Figure 2E**). The expanded CPR cohort confirmed antibody titers persisting for 50 days post third-dose administration, numerically comparable to CR though not statistically superior (**Figure 2G**).

*Pb*SLTRiP proved intrinsically immunogenic, even without adjuvant, with SPR associated with earlier responses and higher IgG2c alongside IgG1, suggesting balanced subclass engagement without uniform endpoint titer superiority (**Figure 4**). SPR showed early titer advantages (**Figure 4**), but C57BL/6 mice naturally exhibit Th1-biased responses with preferential IgG2c production even in the absence of Th1-promoting adjuvants (38–40). Consequently, IgG2c detection largely reflects this strain-specific baseline rather than conclusive evidence of TLR7/8-mediated skewing. SPR was associated with higher response magnitude against this intrinsic background. The avidity indices showed increasing trends under SPR conditions (**Supplementary Figure S5**), potentially reflecting affinity maturation processes.

*Pf*CSP immunization with antigen alone primarily produced IgG1 responses in Th2-prone BALB/c mice, whereas the dual-adjuvant CPR formulation produced both IgG1 and detectable IgG2a at earlier time points, suggesting broader isotype engagement (**Figure 3**). No significant differences in IgG1 titer were observed between the CSP-only and CPR groups (**Figure 2C**), and ELISA endpoint measurements alone do not fully capture dual adjuvant contributions to humoral immunity quality. Complementary functional studies, including B-cell subset analysis, avidity maturation assessment, and effector function evaluation, are essential, particularly given our *in silico* predictions of synergistic TLR3/7–8 signaling and multi-epitope vaccine design optimization. Similarly, *Pb*SLTRiP and SPR regimens produced combined IgG1/IgG2c responses (**Figure 4**), consistent with potential engagement of multiple helper T-cell pathways despite the absence of formal Th1/Th2 polarization assessment. The pronounced IgG2a/c responses are consistent with IFN-γ-driven class switching, supporting T-cell-dependent pathways that contribute to humoral immunity (40–42). Collectively, these observations suggest that dual TLR agonist formulations may enhance the breadth and durability of antibody responses in comparison to the unadjuvanted controls. The larger cohort studies are indeed required for the confirmation and for statistically significance. When considered alongside current pre-erythrocytic vaccine platforms, such as RTS,S/AS01 (Mosquirix) (43) and R21/Matix-M (44), both rely on saponin-based adjuvants that potently stimulate innate immunity but still show waning efficacy over time (13, 45). This underscores the need for adjuvant systems capable of mounting sustain responses. Poly(I:C) and R848 engage TLR3 and TLR7/8 pathways, reported to promote B-cell maturation and antigen presentation. In-depth investigation is required to determine whether TLR3 and TLR7/8 engagement may support durable antibody responses against *Pf*CSP and *Pb*SLTRiP. Since the present study focused on the serological readouts and used non-parametric tests, it did not show broad, statistically significant superiority of the combination of adjuvants over the single-adjuvant regimens. We suggest investigations with a larger animal cohort to confirm that the dual TLR targeting may offer an advantage in the magnitude, durability, or functionality of the humoral immune response.

The infection-cure model experiments demonstrate the biological relevance of *Pb*SLTRiP as an *in vivo* target. In the CQ-infection/cure model, repeated SPZ exposure under CQ cover elicited detectable *Pb*SLTRiP-specific IgG responses, which were boosted by subsequent unsuppressed challenge infections and remained elevated across multiple pre-erythrocytic exposures (**Figure 5**). Because *Pb*SLTRiP is expressed in both SPZ and LS parasites, the antigen is naturally encountered by the host immune system during infection and may elicit recall antibody responses, advocating its potential as a “vaccine candidate”.

The epitope summary (**Figure 6A**) also reveals multiple predicted B-cell, CD4, and CD8 T-cell epitopes in *Pf*CSP, *Pb*SLTRiP, and *Pf*SLTRiP antigens, whereas CD8 and CD4 T-cell predictions identify regions with high predicted MHC I and II binding affinity that overlap with putative B-cell epitopes (**Supplementary Figure S6**).

*In silico* epitope analysis (**Figure 6A**) identified overlapping B- and T-cell epitopes that provide a rationale to explore humoral-cellular immunity mechanisms. The conserved interfaces in Poly(I:C)/R848 suggest potential for multi-pathway TLR engagement. Recent work by Gao et al. offers conceptual support for this hypothesis, as their study showed that B cells targeting *Plasmodium* CSP may capture not only their cognate antigen but also spatially linked surface antigens from the parasites (46). This suggests that B cells may help CD4^+^ T cells recognize either the same or adjacent antigens, thereby broadening and enhancing humoral immunity.

The combination of adjuvant formulations showed trends toward sustained IgG titers, increased avidity, and broader IgG subclass profiles compared to those seen with antigen devoid of adjuvants. No statistical significance was observed, likely reflecting assay variability or antigen-dosing limitations. Future nanoparticle optimization could enhance these trends for malaria antigens (47).

The advanced preclinical models (48–50), such as humanized mice, may also be used to explore Poly(I:C)+R848 adjuvant combinations and multi-subclass antibody responses in the human settings, paving the way for clinical investigation.

## Data Availability

The raw data supporting the conclusions of this article will be made available by the authors, without undue reservation.
