# Supplementary material for: Poly(I:C) and R848 adjuvants elicit sizeable humoral immunity to liver stage malaria antigens
Source: Front Immunol. 2026 Mar 30;17:1705839. doi: 10.3389/fimmu.2026.1705839 (PMC13070761; doi:10.3389/fimmu.2026.1705839)
Supplement: Supplementary file 1 [file DataSheet1.docx]

**SI Information**

**Repeated immunization of *Pf*CSP in antigen-alone and different combinational approach of adjuvant enhances *Pf*CSP-specific IgG response**

**SI Figure 1. The increased and functionally balanced humoral response to *Pf*CSP with dual TLR adjuvant regimen (A-D)** *Pf*CSP-specific total IgG kinetics following repeated immunization under different adjuvant conditions. Line graphs show ELISA titration curves for serum total IgG in mice immunized with *Pf*CSP only **(A),** *Pf*CSP+Poly(I:C) (CP) **(B)**, *Pf*CSP+R848 (CR) **(C)**, and *Pf*CSP+Poly(I:C)+R848 (CPR) **(D)**. Responses were monitored after the 1st, 2^nd^, 3^rd^ doses, and 50 days post-3^rd^ dose (durability). n=3-4 mice/group for single adjuvant and initial pilot studies). Data are presented as mean±SEM.

**SI Figure 2. *Pf*CSP-specific IgG subclass kinetics after repeated immunization with CSP protein alone/CPR.** (A-B) *Pf*CSP-specific IgG subclass responses in mice immunized with *Pf*CSP only. Line graphs show ELISA titration curves for IgG1 **(A)** and IgG2a **(B)** after successive immunization. **(C-D)** *Pf*CSP-specific IgG subclass responses in mice immunized with CPR regimen. Line graphs show ELISA titration curves for IgG1 **(C)** and IgG2a **(D)** after successive immunization, including a 50-day post-3^rd^ dose time point demonstrating sustained functional balance.

***Pb*SLTRiP protein expression, purification and immunization**

For protein expression, a primary culture was prepared by inoculating from the stock culture in LB broth containing 100 μg/mL ampicillin. A parallel, uninoculated flask served as a control. Both flasks were incubated overnight at 37°C with shaking at 150-180 rpm. The overnight culture was then expanded to a total volume of 1 L (secondary culture) and incubated at 37°C with shaking. Upon reaching an optical density at 600 nm (OD600) of 0.6-0.7, protein expression was induced by the addition of IPTG to a final concentration of 1mM. IPTG addition and subsequent incubation were performed in the dark. The cultures were then incubated for approximately 16 h at 37°C with shaking at 200-220 rpm. Following expression, cells were harvested by centrifugating at high speed for 30-45 minutes at 4**°**C. After centrifugation, the supernatant was discarded, and the cell pellet was resuspended in lysis buffer. The cell pellet was lysed with 10-20 mL of lysis buffer per liter of original culture. This process was repeated as necessary to maximize cell recovery. The lysate was then sonicated using a LABMAN Probe Sonicator with a 3 mm diameter probe (1 second ON, 2 seconds OFF) for a total of 50 minutes, or until the lysate exhibited a free-flowing consistency. The resulting lysate was filtered through a 0.45 μM nylon syringe filter (AXIVA, 33 mm diameter). The filtrate was applied to a GSTrap FF 5 mL column (GE Healthcare), previously activated/equilibrated with 5 column volumes of binding buffer. The sample was loaded manually using a 5 or 10 mL syringe at a flow rate of 1-5 mL/min. To ensure maximal binding, the flow-through was reapplied to the column. The column was then washed with ̴ 35 mL of binding buffer, followed by 35 mL of binding buffer containing 2 mM reduced glutathione. Finally, the protein was eluted with 3-5 column volumes of elution buffer. Eluted fractions were collected in 1 mL Eppendorf tubes and stored at -20°C and run on the SDS-PAGE (**SI Fig. 3A**).

**SI Figure 3. *Pb*SLTRiP recombinant protein characterization and humoral immune response kinetics, and isotypes following immunization with/without adjuvants. (A) SDS PAGE analysis of the recombinant *Pb*SLTRiP protein.** The antigen was expressed, purified via GST-affinity chromatography, and resolved in the distinct fractions by the SDS-PAGE and resolved 75 kDa (*Pb*SLTRiP-GST fusion), 47 kDa (GST), and 25 kDa (*Pb*SLTRiP). **(B) Immunization strategy scheme** illustrating the dosing regimen for the *Pb*SLTRiP antigen in C57BL/6 mice (three subcutaneous doses administrated every 14 days) and the time of blood collection similar to that one with *Pf*CSP antigen. **(C-F)** Humoral immune response elicited by *Pb*SLTRiP without adjuvants line graphs show ELISA titration curves for serum antibody levels-total IgG (C), IgG1 (D), IgG2c (E), and IgM (F)-following 1^st^, 2^nd^, and 3^rd^ dose. **(G-J)** Humoral immune response elicited by *Pb*SLTRiP with dual TLR adjuvants (Poly(I:C) and R848) (SPR). Line graphs show ELISA titration curves for serum antibody levels**-**-total IgG (G), IgG1 (H), IgG2c (I), and IgM (J)-with the 30 days post-3^rd^ dose time point demonstrating durability. The *Pb*SLTRiP only group contains n=3-4 mice/group, while the SPR adjuvanted group (Panels G-J) represents the pooled data from two independent experiments (n=4 mice/group). Data are presented as mean ± SEM.

**Assessment of GST-Tag–Induced Antibody Response**

The recombinant *Pb*SLTRiP protein was expressed with an N-terminal Glutathione S-transferase (GST) tag, which remained uncleaved prior to immunization. To definitively attribute the observed immune response to the *Pb*SLTRiP protein rather than the GST-tag, a dedicated control experiment was performed. 3-4 C57BL/6 mice were immunized under identical experimental conditions, with purified GST protein serving as the immunogen *in lieu* of the *Pb*SLTRiP-GST fusion protein. Subsequently, an indirect ELISA was performed to evaluate the elicited antibody response against the *Pb*SLTRiP protein. The results demonstrated that the GST-tag alone did not induce a statistically significant antibody response above baseline levels. Any negligible signal detected may be attributable to a non-specific immunostimulatory effect of the adjuvants employed in the immunization protocol, suggesting a potential for minimal adjuvant-induced background reactivity in the absence of a specific antigen (**SI Fig. 4**). This control experiment unequivocally establishes that the humoral immune response observed in the primary study was specific to the *Pb*SLTRiP protein and not an artifact of the GST-tag.

**SI Figure 4:** **The antibody response following the repeated immunizations with GST only alongside adjuvants.** The data represented is the pool of two independent experiment (n=3-4 mice/group). Data are presented as mean±SEM.

**SI Figure 5. *Pb*SLTRiP recombinant protein avidity following immunization with/without adjuvants****. (A-B)** Durability and functional quality of the *Pb*SLTRiP only group. Scatter plot **(A)** shows endpoint titers following each dose. Bar graph **(B)** shows the antibody avidity index (AI) following the 1st, 2^nd^, and 3^rd^ doses. Line graph (M) show ELISA curve for AI using urea solution. **(C-D)** Durability and functional quality of the SPR group. Bar graph **(C)** shows the AI confirming affinity maturation over time. Line graph **(D)** show ELISA curve for AI calculation, confirming enhanced binding strength across PBS and urea elution. Data are presented as mean±SEM. The *Pb*SLTRiP only group contains n=3-4 mice/group, while the SPR adjuvanted group (Panels C-D) represents the pooled data from two independent experiments (n=4 mice/group). Difference in titers across time points within each immunization group were analyzed using the Kruskal-Wallis test followed by Dunn’s post-hoc multiple-comparison procedure, with significance denoted as ns (not significant), * p < 0.05, ** p < 0.01, *** p < 0.001, **** p < 0.0001. AI was calculated at dilutions chosen to approximate similar mean PBS OD values across doses; small deviations in OD between doses may contribute to minor non‑monotonic changes in AI

1. ***Plasmodium falciparum CSP***

MMRKLAILSVSSFLFVEALFQEYQCYGSSSNTRVLNELNYDNAGTNLYNELEMNYYGKQENWYSLKKNSRSLGENDDGNNEDNEKLRKPKHKKLKQPADGNPDPNANPNVDPNANPNVDPNANPNVDPNANPNANPNANPNANPNANPNANPNANPNANPNANPNANPNANPNANPNANPNANPNANPNANPNANPNVDPNANPNANPNANPNANPNANPNANPNANPNANPNANPNANPNANPNANPNANPNANPNANPNANPNANPNANPNKNNQGNGQGHNMPNDPNRNVDENANANSAVKNNNNEEPSDKHIKEYLNKIQNSLSTEWSPCSVTCGNGIQVRIKPGSANKPKDELDYANDIEKKICKMEKCSSVFNVVNSSIGLIMVLSFLFLN

1. ***Plasmodium berghei SLTRiP***

MYVYVTLKFIKNSFHVGKIINSKKQHSNLQYISYIFIYFFIFPSIPLYKKILYSNVKHRLKTFLFMPESKIIQWKQKGENSDDEYSDDEYSDEDQYNNEENSENEYTDGEITETEEREDKNLIAGTNSNKSPKNVSVNNDTTRLDKTGGKICYFFDKKKTSYRKLTEYWKRRRWKKYLKKVDNEWQLLNLGIENVIKKMIERNNAELEKWKTQQAHKWLHSNNLYAQYASLYKKITPSIEIDNVIEQMGNKLKEKIYNRWNNLQTENENNIRKWVIEQWNEWKNAKIISWLMCDWKRNENEKWVQWKNKYRYHIKYAPNRNEYHVWQKRTNIEKKQWSNWVRIKEDHYIYNIEILCNKEKNAYKKSIIKWINDIADNFVKNPQLRLWIEQQVKPFPRKKLLKGSKVKSIESSNIEINA

1. ***Plasmodium falciparum SLTRiP***

MNLEQFKNINKDLATNLFSQLSFLKNENKFLSQGKSLIKFLIGIAIFLVVLIFIKSSHPALKEKKKKVLEFFENLVLNKKKKENITAAIASKELADAETTDTSDSEDEDHIINKKVKRRKRNIINNPDEKVHNVKEKNSKSKNEEDKTDESYNETSLLSSDEEGEVNLEDWKKNEWIKWMDETEEEWQLLKLWLEGEKNKWLEGKNKEYDIWLNHMNSKWTNYNKDIDEEYDSNVFKDSYKWNEKQWEQWMKTEGKEFMLQDFKRWLEDSEGYLKSWLIKQWIQWKNMKILECLMNEWRREEDEKWSNLEDTDQIRVLNHKDRKEWLRWKERVTREKLEWKHWVEMKENMNIYNKWKKWIKWKKNKLANFNEWSKNFIEKWIREKQWNNWINERKKYTSQRKSLEQQFGDNMDKMNKLKKKKILKFFPLFNYKSDLESIMEEDENEYNSFDENEEENDEKTGDVNVGKTEALNVAKTEGLNVGKTEDLNVAKTEDLNVAKTADLNAEKTTDLNSEKTADLNSDKTTDLNPEKTTNFNTYKATDLNANKTADLNSDKTTDLNSDKTTNFNTYRTTDLNSDKTTNFNTYKTDLYAEKTTDVNLGKTTNHNVAKTTDQKVVKHSLDHEVRQMIDQKVAQIMNHDLESTAEQKAEKKGGKAKAKTKVRTVDDDGNEINV

**B-Cell Epitope CD8 T Cell Epitope CD4 T Cell Epitope**

**SI Figure 6:** ***In-silico* identification of B cell, CD8^+^ and CD4^+^ T cell epitope using IEDB for CSP and SLTRiP malaria liver-stage protein antigens.** With the usage of Bepipred Linear Epitope Prediction 2.0, NetMHCpan 4.1 EL and NetMHCIIpan 4.1 EL of IEDB tool, epitopes were identified for **(A)** CSP of *P. falciparum*, and SLTRiP of **(B)** *P. berghei, and* ***(C)*** *P. falciparum*. The parameters were set to the default throughout the epitope identification. Further, H-2K^b^ or H-2D^b^, and H-2K^d^ or H-2D^d^ have been used (peptide length=8/9 mer) for CD8^+^ T cell epitope identification in C57BL/6 (for SLTRiP), and Balb/C (for CSP) mice, respectively.

**SI Figure 7: *Molecular interactions in dual adjuvant-TLR complexes:* (A)** TLR3–poly(I:C) complex (PDB 7WV5). Left: TLR3 ectodomain dimer (Chain A = red, Chain B = yellow) bound to poly(I:C) dsRNA (Chain E = blue, Chain F = black). Right: Close-up showing hydrogen bonds (green), salt bridges (red) and polar contacts (blue) between TLR3 basic residues and the dsRNA phosphate backbone **(B)** TLR8–R848 complex (PDB 3W3M). Left: TLR8 ectodomain (green) bound to R848 (yellow). Right: Close-up showing hydrogen bonds (green), hydrophobic contacts (red), and π-π stacking (black) within the ligand-binding pocket.
Visualized using BIOVIA Discovery Studio Visualizer.

**SI Figure S8. Structural conservation of human and murine TLR7/8**. **(A)** Crystal structure of human TLR7 ectodomain in complex with the small‑molecule agonist Cpd‑1 (SM‑374527), shown as a horseshoe‑shaped LRR scaffold with helices (red), strands (cyan), and loops (grey). **(B)** Crystal structure of human TLR8 ectodomain in complex with the imidazoquinoline agonist Resiquimod (R848), highlighting the circular LRR arrangement and bound ligand within the central pocket. **(C)** Structural superposition of human TLR7 (6LVX, one colour) and the homology model of mouse TLR7 ectodomain (second colour), illustrating preservation of the overall LRR architecture and ligand‑binding groove. **(D)** Structural superposition of human TLR8 (3W3M) and the homology model of mouse TLR8 ectodomain, showing close overlap of the ectodomain scaffold and conservation of the agonist‑binding pocket.
